# Comparative Genomics Reveals Evolutionary Drivers of Sessile Life and Left-right Shell Asymmetry in Bivalves

**DOI:** 10.1016/j.gpb.2021.10.005

**Published:** 2022-01-26

**Authors:** Yang Zhang, Fan Mao, Shu Xiao, Haiyan Yu, Zhiming Xiang, Fei Xu, Jun Li, Lili Wang, Yuanyan Xiong, Mengqiu Chen, Yongbo Bao, Yuewen Deng, Quan Huo, Lvping Zhang, Wenguang Liu, Xuming Li, Haitao Ma, Yuehuan Zhang, Xiyu Mu, Min Liu, Hongkun Zheng, Nai-Kei Wong, Ziniu Yu

**Affiliations:** 1 CAS Key Laboratory of Tropical Marine Bio-resources and Ecology and Guangdong Provincial Key Laboratory of Applied Marine Biology, Innovation Academy of South China Sea Ecology and Environmental Engineering, South China Sea Institute of Oceanology, Chinese Academy of Sciences, Guangzhou 510301, China; 2 Southern Marine Science and Engineering Guangdong Laboratory (Guangzhou), Guangzhou 511458, China; 3 Biomarker Technologies Co., Ltd, Beijing 101301, China; 4 CAS Key Laboratory of Experimental Marine Biology, Center for Mega-Science, Institute of Oceanology, Chinese Academy of Sciences, Qingdao 266071, China; 5 State Key Laboratory of Biocontrol, College of Life Sciences, Sun Yat-sen University, Guangzhou 510275, China; 6 Zhejiang Key Laboratory of Aquatic Germplasm Resources, College of Biological and Environmental Sciences, Zhejiang Wanli University, Ningbo 315100, China; 7 College of Fisheries, Guangdong Ocean University, Zhanjiang 524088, China; 8 Hebei Key Laboratory of Applied Chemistry, College of Environmental and Chemical Engineering, Yanshan University, Qinhuangdao 066044, China; 9 Department of Pharmacology, Shantou University Medical College, Shantou 515041, China

**Keywords:** Comparative genomics, Ostreoida oyster, Attachment, Shell asymmetry, Bivalve

## Abstract

**Bivalves** are species-rich mollusks with prominent protective roles in coastal ecosystems. Across these ancient lineages, colony-founding larvae anchor themselves either by byssus production or by cemented **attachment**. The latter mode of sessile life is strongly molded by left-right **shell asymmetry** during larval development of **Ostreoida oysters** such as *Crassostrea hongkongensis*. Here, we sequenced the genome of *C. hongkongensis* in high resolution and compared it to reference bivalve genomes to unveil genomic determinants driving cemented attachment and shell asymmetry. Importantly, loss of the homeobox gene *Antennapedia* (*Antp*) and broad expansion of lineage-specific extracellular gene families are implicated in a shift from byssal to cemented attachment in bivalves. Comparative transcriptomic analysis shows a conspicuous divergence between left-right asymmetrical *C. hongkongensis* and symmetrical *Pinctada fucata* in their expression profiles. Especially, a couple of orthologous transcription factor genes and lineage-specific shell-related gene families including that encoding tyrosinases are elevated, and may cooperatively govern asymmetrical shell formation in Ostreoida oysters.

## Introduction

Bivalves belong to the ancient lineages of Mollusca, comprising nearly 9600 species that thrive in aquatic environments, with notable economic and ecological importance [1]. As bilaterian organisms, they rely nutritionally on filtering phytoplankton and primarily follow a life cycle that transitions from free-swimming larvae to attached juveniles, culminating in sessile life [2]. Among filter-feeding bivalves, oysters of the superfamily Ostreoida serve as crucial guardians of marine ecosystems by forming oyster reefs that clean up the water and sustain biodiversity [3]. Due to climate change and coastal degradation, however, bivalves face profound challenges from warming waters and ocean acidification, which destabilize habitats, raise infection risks, and dampen the bivalve capacity of acquiring carbonate for shell formation [4].

To cope with diverse ecosystems, a variety of sessile strategies have emerged in bivalves during evolution, among which two modes of sessile life prevail. Characteristically, the majority of the bivalves, including Mytilidae (mussel), Pectinidae (scallop), and Pteriidae (pearl oyster), secrete adhesive byssal threads to stabilize themselves against marine turbulences [5], [6]. In contrast, Ostreoida oysters have evolved highly sophisticated machinery of cemented attachment by producing organic-inorganic hybrid adhesive substances in place of byssus, which allows them to permanently fuse the left shell with rock surfaces or shells of other individuals in intertidal zones [7]. Compared with byssus, cemented attachment exhibits superiority in physical adhesion and mechanical tension, enabling oysters to efficiently create and thrive in large reef communities [2]. Developmentally, as a salient feature of their exoskeleton, shell formation processes in bivalves are strongly molded by their preferences for sessile life [8]. Quite distinctively, byssally attached bivalve species tend to possess a bilaterally symmetrical shell, whereas cement-attached oysters present a high degree of phenotypic variability and morphological asymmetry characteristic of their radically distinct left-right (L/R) shells [8]. Nevertheless, the molecular mechanisms driving these extraordinary innovations in bivalve evolution remain enigmatic, particularly in genomic contexts.

The Hong Kong oyster (*Crassostrea hongkongensis*, first described as *Crassostrea rivularis* by Gould, 1861) is an economically valuable aquacultural species endemic to the South China coastline [9]. As an ideal model for studying shell asymmetry, *C. hongkongensis* larvae follows a typical developmental cycle of cemented attachment and asymmetrical differentiation of the L/R shells. In order to elucidate the genetic basis underpinning the evolution of bivalve sessile life and asymmetry of shell formation, we sequenced and analyzed the complete genome of *C. hongkongensis* and performed comparative genomic analysis along with several other bivalve species, including two congeneric Ostreoida oysters, *Crassostrea gigas* (Pacific oyster) and *Crassostrea virginica* [6], [10], [11], [12]. In addition, we monitored the transcriptomic changes of *C. hongkongensis* embryos during the critical window of larval attachment, and compared the expression patterns of asymmetry-related genes in the L/R mantles of adult *C. hongkongensis* and byssus-producing pearl oyster (*Pinctada fucata*). Our comparative genomic data and associated functional assays reveal extensive molecular adaptations across the oyster genome that support the evolutionary switch from byssal to cemented attachment and divergence from the symmetrical shell in Ostreoida oysters.

## Results and discussion

### Genome assembly and evolutionary analysis of *C. hongkongensis*

**Supplementary figure S1:**
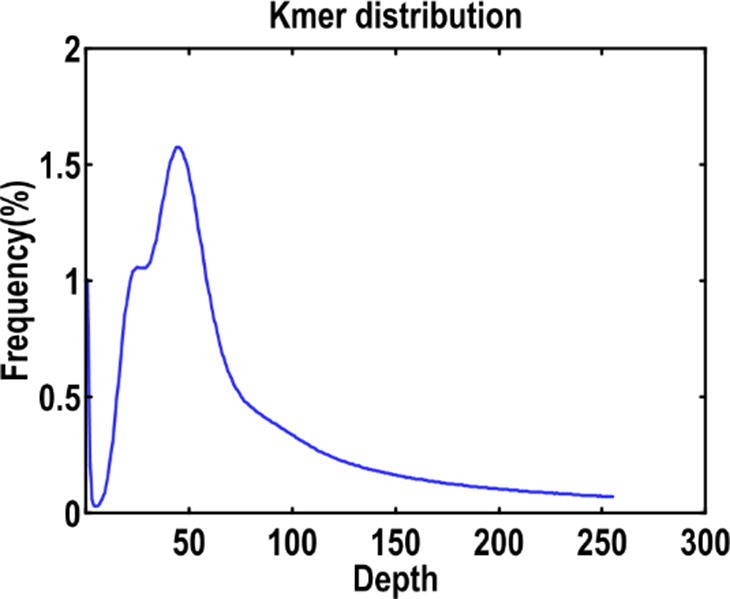
**Seventeen k-mer depth distribution of Crassostrea hongkongensis genome sequences**. Depth distribution of k-mer showed a major peak at 45×, and genome size was estimated to be ∼ 593 Mb according to the formula: Genome size= k-mer number/peak depth.

Efforts on genome sequencing and assembly are inherently challenging for many marine invertebrates such as mollusks, annelids, and platyhelminths due to their remarkable genetic heterozygosity (or polymorphisms) [10], [11], [13]. Based on *k*-mer analysis, the genome size of a single wild-stock Hong Kong oyster (*C. hongkongensis*) individual was estimated to be 695 Mb with 1.2% of heterozygosity (Figure S1), comparable to that of the Pacific oyster (1.3%) [10]. To circumvent limitations of short-read next-generation sequencing in assembling highly polymorphic genomes, PacBio sequencing combined with Illumina sequencing was used as the dominant mode of genome sequencing in our study. We first generated 23.25 Gb of raw PacBio reads and 147.25 Gb of Illumina reads, being equivalent to 31.9× and 201.8× genome coverages, respectively (Tables S1 and S2). Following stepwise optimization of assembly algorithms, these reads were assembled into a 729.6-Mb genome with a contig N50 size of 314.1 kb, a scaffold N50 size of 500.4 kb, and the longest contig spanning 2.37 Mb (Table S3). The contig N50 size of the oyster genome is at least one order of magnitude more expansive than those of published bivalve genomes (Table S4), demonstrating the superiority of long-read sequencing technologies in coping with high polymorphism in genome assembly of marine invertebrates. However, the assembled genome size was slightly larger than that estimated by *k*-mer analysis. Such discrepancy may reflect sequence preferences of Illumina reads. The high integrity and quality of the assembly were evidenced by a productive mapping of 97.57% of sequencing reads and a low single-nucleotide error rate (Tables S5 and S6). Moreover, benchmarking universal single-copy orthologs (BUSCO) analysis confirmed a high degree of completeness (92.84%) for the assembled genome (Table S7), which is comparable to genome completeness of other published bivalves (Table S4).

**Figure 1:**
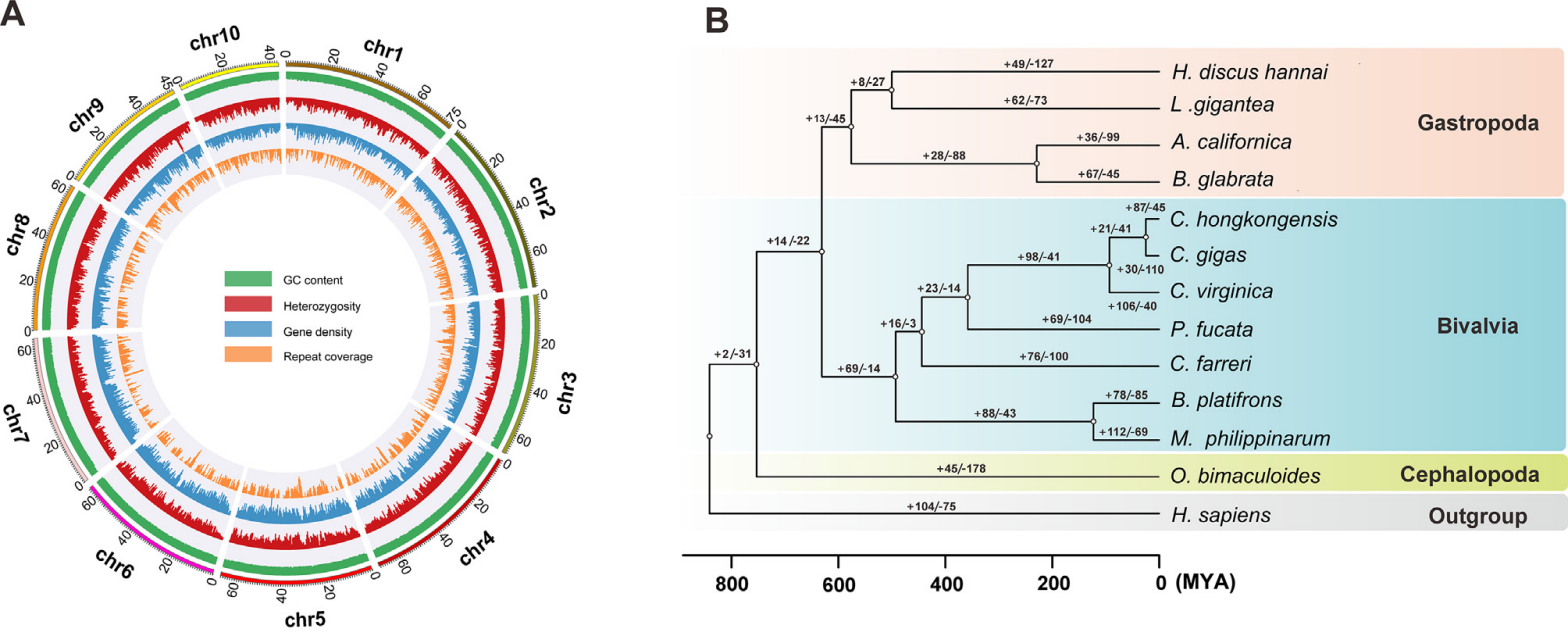
**The genome landscape and phylogenetic analysis of the oyster *C*.*hongkongensis*** **A.** Circos plot highlights genome characteristics across 10 chromosomes in a megabase (Mb) scale. The GC content, global heterozygosity, gene density, and repeat coverage are presented from outer to inner circles in turn with non-overlapping 1 Mb sliding windows. **B.** Analysis on gene family expansion/contraction and divergence time across 12 representative mollusk species. A total of 87 gene families were expanded in *C. hongkongensis* (Hong Kong oyster). The human genome was set as outgroup. Three Ostreoida oyster species (*C*. *hongkongensis*, *C*. *gigas*, and *C*. *virginica*) were clustered together. Gene family expansion/contraction is indicated by a plus or minus sign. MYA, million years ago; *H. discus hannai*, *Haliotis discus hannai*; *L. gigantea*, *Lottia gigantea*; *A. californica*, *Aplysia californica*; *B. glabrata*, *Biomphalaria glabrata*; *C. hongkongensis*, *Crassostrea hongkongensis*; *C. gigas*, *Crassostrea gigas*; *C. virginica*, *Crassostrea virginica*; *P. fucata*, *Pinctada fucata*; *C. farreri*, *Chlamys farreri*; *B. platifrons*, *Bathymodiolus platifrons*; *M. philippinarum*, *Modiolus philippinarum*; *O. bimaculoides*, *Octopus bimaculoides*; *H. sapiens*, *Homo sapiens*.

**Supplementary figure S2:**
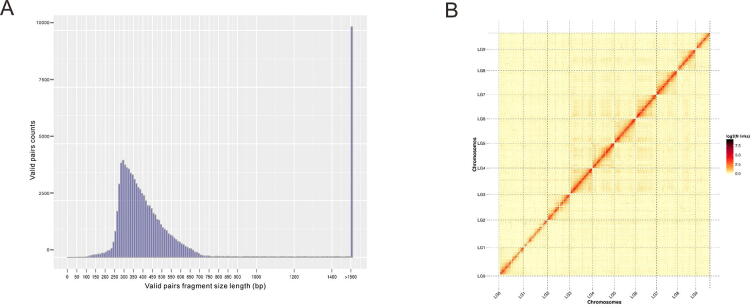
**Count distribution of valid-pairs fragment size**. **A**. A total of 36.82 Gb clean Hi-C reads were aligned to the *C. hongkongensis* genome assembled genome. Valid interaction pairs were defined as unique mapped reads spanning two digested fragments, which were distally located but physically associated DNA molecules. **B**. Chromosomal *C. hongkongensis* genome was assembled with the aid of Hi-C data.

**Supplementary figure S3:**
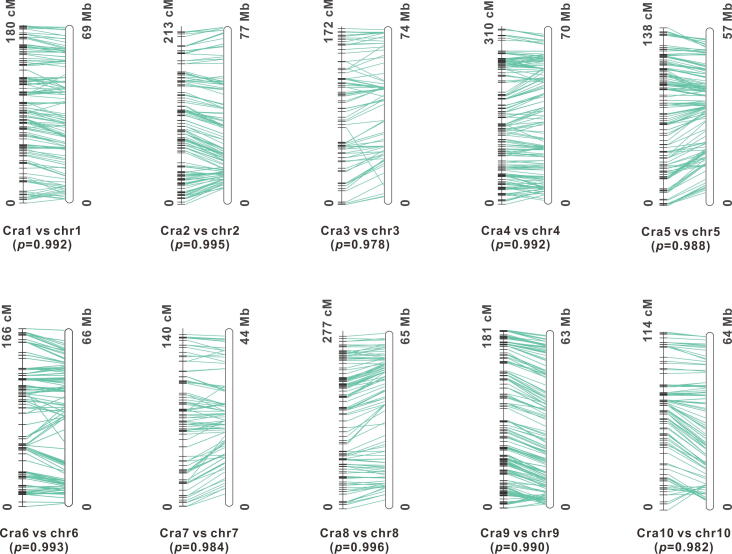
**Consistency between Hi-C data-based pseudo-chromosomes and a genetic map for the relative species, *Crassostrea gigas***. Chr1-10 and Cra1-10 indicate the Hi-C data based pseudo-chromosomes of *C. hongkongensis* and the genetic map for *Crassostrea gigas*, respectively. *P* values refer to their consistency, which was assessed by using the software ALLMAPS.

To assemble the oyster genome to the chromosomal level, we generated ∼ 44.4 million valid Hi-C interaction pairs with over 50× coverage (Table S8). Then, 690.39 Mb of genome sequences were anchored into 10 pseudo-chromosomes with Hi-C data, covering 94.66 % of the assembled genome (Figure 1A, Figure S2; Table S9). Among them, 648.56 Mb of genome sequences were reoriented and anchored into chromosomes, constituting 93.94% of the total anchored sequences (Table S9). Moreover, high consistency between Hi-C-based pseudo-chromosomes with the genetic map of one congeneric species, *C. gigas*, was confirmed (*P* = 0.978–0.996, Figure S3), implicating high reliability in chromosomal genome assembly. Overall, by leveraging PacBio and Hi-C-enhanced Illumina sequencing, a very high-quality and chromosome-anchored complete genome was obtained, thus providing a robust framework for subsequent exploration of oyster biology and evolution of bivalves.

**Supplementary figure S4:**
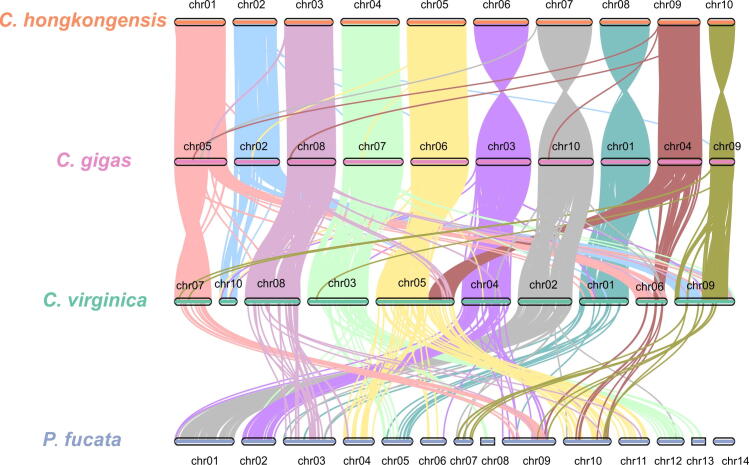
**Genomic synteny between the genomes of three Ostreoida oysters and *Pinctada fucata***. All-to-all BLASTP analyses of protein sequences were set at a threshold of 1E-5. Syntenic regions within and between species were identified by using MCScan based on BLASTP results. A syntenic region was defined as a minimum of 10 collinear genes and a maximum of 25 gaps (genes) between two adjacent collinear genes.

For gene annotation, we predicted 30,021 protein-coding genes in the genome by integrating results from *ab initio* prediction, homology-based searches with reference genomes, and RNA-seq-assisted prediction (Table S10), with an estimated BUSCO completeness of 91.09% (Table S11). Of these, more than 97.97% (28,329/30,021) of the predicted genes were annotated in the public databases (Table S12). The gene number here was similar to that in a close relative species, *C. gigas* (28,027 genes) [10]. In addition, transposable elements (TEs) constituted 46.2% of the *C. hongkongensis* genome, among which the predominant type of TEs was class II Helitron (12.4%, 90.4 Mb) (Table S13). Phylogenetic analysis showed that three Ostreoida oyster species (*C. hongkongensis*, *C. gigas*, and *C. virginica*) clustered together (Figure 1B) and that Ostreoida oyster speciation occurred around 92.1 million years ago (MYA), in agreement with evidence from mitochondrial genomes [14]. Within bivalves, Ostreoida oysters are closest to the Pteriidae oyster *P*. *fucata*, and their divergence time was estimated to be 357.5 MYA (Figure 1B). These results further verified the hypothesis that a common ancestor of primitive Ostreoida and Pteriidae oysters existed prior to the Permian-Triassic extinction event, whereas speciation of modern Ostreoida oysters began at the end of Cretaceous-Paleogene extinction event [15]. Consistently, comparative genomic synteny analysis showed that three Ostreoida oyster genomes had high genomic collinearity except for large intra-chromosomal inversions, but substantial inter-chromosomal translocations and rearrangements occurred between chromosomes of Ostreoida oysters and *P*. *fucata* (Figure S4), in agreement with their phylogenetic relationships and divergence time.

### Loss of the ***Antennapedia*** gene in Ostreoida oysters

**Figure 2:**
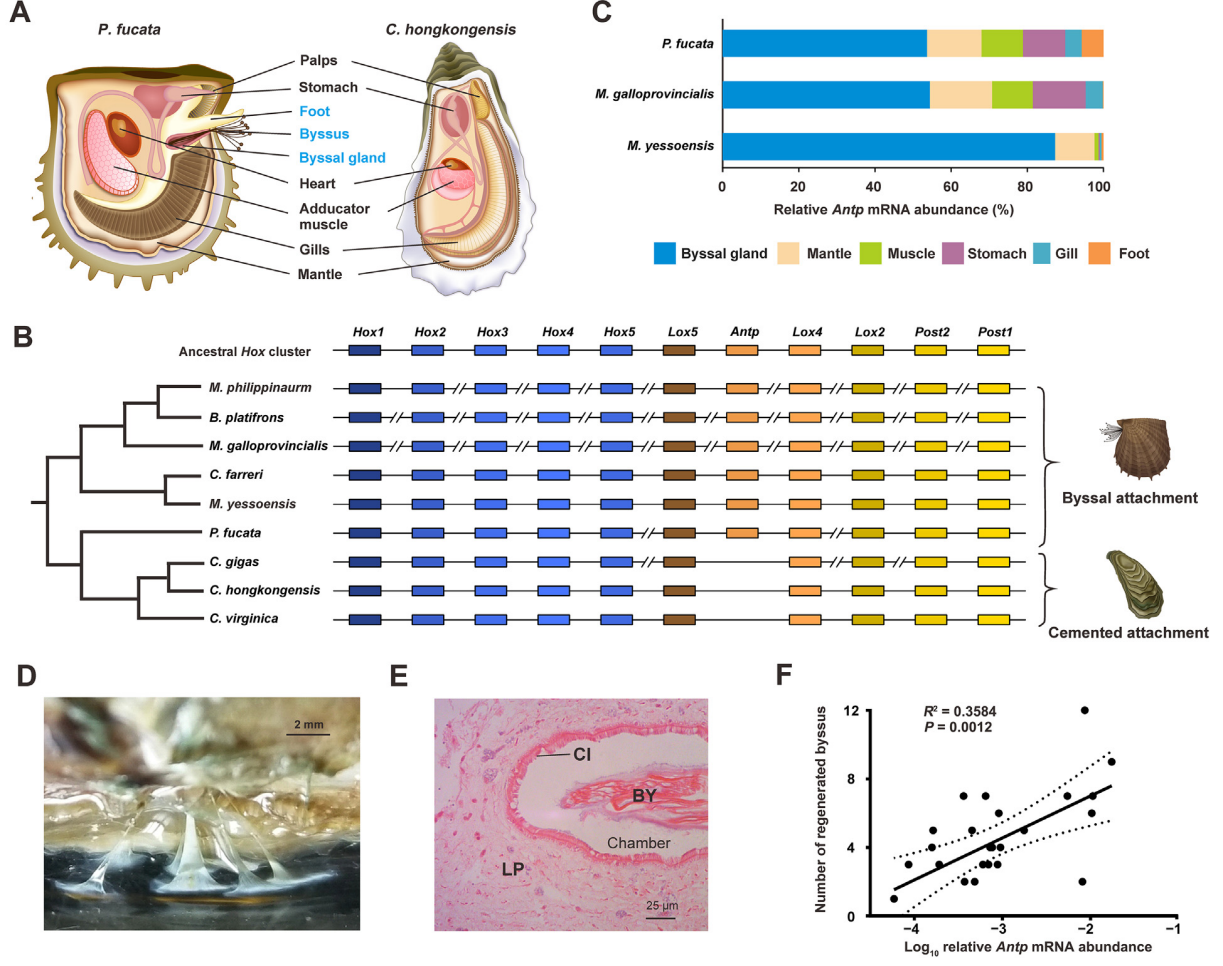
**Loss of the*****Hox*****gene*****A******ntennapedia* is implicated in an adaptive shift from byssal attachment to cemented attachment** **A.** Overview of the key body-plan organization in *P. fucata* and *C. hongkongensis*. *P. fucata* possesses a byssal gland and byssus, whereas adult individuals of Ostreoida oyster have lost their byssus gland and byssus. **B.** Comparison of the *Hox* cluster organization in bivalves with two distinct attachment styles, byssal attachment and cemented attachment. Unlike the disputed *Hox* gene cluster in *C. gigas* oyster genome, the *Hox* gene cluster is configured linearly in both *C. hongkongesis* and *C. virginica*. Intriguingly, *Antp* is lost in all three Ostreoida oysters. **C.** Tissue distribution of *Antp* orthologues in three byssally attached bivalves, *P. fucata*, *M*. *galloprocincialis*, and *M*. *yessoensis*. *Antp* mRNA abundance is displayed in percentage, and its expression in byssal gland accounts for more than 50%. **D.** Morphology of newly regenerated byssus 48 h after excision of original byssus. Scale bar, 2 mm. **E.** Anatomic analysis of the byssal gland of *P. fucata* with cross section. Vertical cross section of the byssal gland displaying Cl, LP, and BY within a chamber. Scale bar, 25 μm. **F.** Correlation between the relative *Antp* mRNA abundance and the number of regenerated byssus in *P. fucata*. The relative *Antp* mRNA abundance in the byssal gland was determined by real-time qPCR, while newly regenerated byssal threads were counted 48 h after excision of original byssus. Pearson’s correlation coefficients and *P* values were calculated using two-tailed tests with 95% confidence. *Hox*, homobox; *Antp*, *Antennapedia*; *M*. *galloprocincialis*, *Mytilus galloprocincialis*; *M. yessoensis*, *Mizuhopecten yessoensis*; Cl, ciliated wall; BY, byssal remnant; LP, lamina propria.

**Supplementary figure S5:**
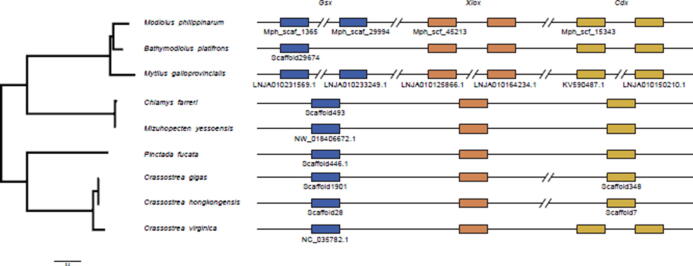
**Evolution and organization of the paraHox gene cluster in bivalves**. A paraHox cluster is composed of three genes, *Gsx, Xlox, and Cdx*. Schematic representation of paraHox genomic evolution and organization in several bivalves: *Modiolus philippinarum, Bathymodiolus platifrons, Mytilus galloprorinclas, Mytilus galloprorinclas, Mizuhopecten yessoensis, Pinctada fucata, Crassostrea gigas, Crassostrea hongkongensis*, and *Crassostrea virginica*. Boxes indicate paraHox genes in the genome. A continuous line indicates an intact cluster. Double-parallel lines indicate long genomic distances.

**Supplementary figure S6:**
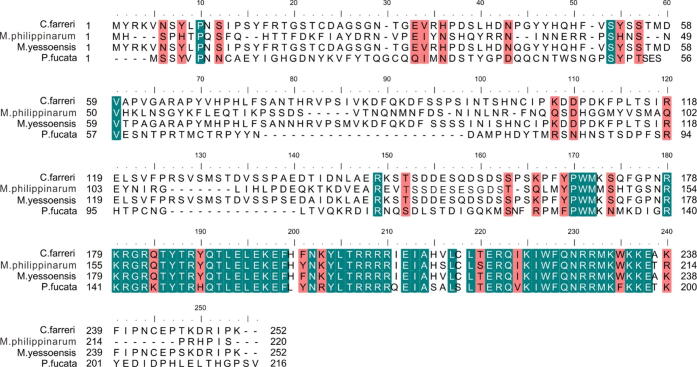
**Sequence alignment of antennapedia (*Antp*) amino acid sequences**. Amino acid sequences of four byssally attached bivalve species (*Chlamys farreri, Modiolus philippinarum, Mizuhopecten yessoensis*, and *Pinctada fucata*) were obtained and aligned by using the Bioedit software. A conserved region with high identity, the Hox domain, can be observed in these four byssally attached bivalve species, which are known to be involved in transcriptional regulation of key developmental processes. In the figure, red color marks the sites with high sequence similarity, while green marks the sites with the same sequence.

Radical changes toward a sessile life require evolutionary innovations in the anatomical organization. In contrast to byssus-producing bivalves [6], Ostreoida oysters do not possess a byssal gland or secrete byssus during their lifetime [16], though a vestigial foot transiently appears at the veliger stage and degenerates following attachment and metamorphosis (Figure 2A). Developmentally, the homeobox (*Hox*) genes are known for their crucial roles in regulating body-plan development and organogenetic transitions in metazoans [17]. In view of this, we compared the clustering of *Hox* genes in byssus-producing and byssus-null bivalve species. A salient feature in byssal bivalves including *P*. *fucata*, *Mizuhopecten yessoensis*, *Chlamys farreri*, *Mytilus galloprovincialis*, *Bathymodiolus platifrons*, and *Modiolus philippinarum* is the intact *Hox* and *ParaHox* gene clusters (Figure 2B, Figure S5). In contrast, a disputed *Hox* gene cluster has been observed in *C. gigas* oyster genome [10], whereas a coherent *Hox* gene cluster is configured linearly in one single locus in both *C. hongkongesis* and *C. virginica*, probably in part due to the fragmented genome assembly in *C. gigas*. Intriguingly, one of the key *Hox* members, *Antennapedia* (*Antp*), is lost in all three Ostreoida oysters (Figure 2B), implicating *Antp* as an essential driver of byssus formation. Sequence alignment reveals that *Antp* possesses a conserved homeobox domain in bivalves (Figure S6).

As evidenced in the expression profiles of three representative byssus-producing bivalve species, *Antp* and its orthologues are predominantly expressed in the byssal gland (Figure 2C). Due to the unavailability of molecular tools like clustered regularly interspaced short palindromic repeat-associated protein-9 (CRISPR/Cas9) or transcription activator-like effector nucleases (TALEN) for manipulating bivalve genomes, genetic ablation of the *Antp* gene is not yet feasible in pearl oyster for phenotypic appraisal of its function. However, histological evidence suggests that byssal gland is one of the appendage organs capable of secreting thin extended byssal threads in their mature form as observable bysuss outside the organism (Figure 2D and E). Based on the fact that regenerative ability varies among individuals, we assessed *Antp* function in this phenotypic trait. Remarkably, mRNA expression levels of *Antp* are highly correlated with the number of regenerative byssus in the pearl oyster (*n* = 24, *R*^2^ = 0.3584, *P* = 0.0012; Figure 2F). Taken together, our evidence strongly implicates that *Antp* is a transcriptional regulator central to byssal secretion in *P. fucata*. Further, the loss of the *Antp* gene seems to be associated with a physical loss of byssal gland in oysters. From an evolutionary perspective, *Antp* seems to play a critical role in appendage diversification in arthropods, which has previously been evidenced by its involvement in the leg formation in the crustacean *Daphnia* [18], and in the repression of the abdominal limb in the spider *Achaearanea tepidariorum* [19]. In addition, ectopic expression of *Antp* in the silkworm (*Bombyx mori*) induced the expression of the *sericin-1* gene in the posterior silk gland [20]. Collectively, these findings support a conserved function of Antp in secretory appendage in two distinct lineages, mollusks and arthropods.

### Gene expansion and oyster attachment

**Figure 3:**
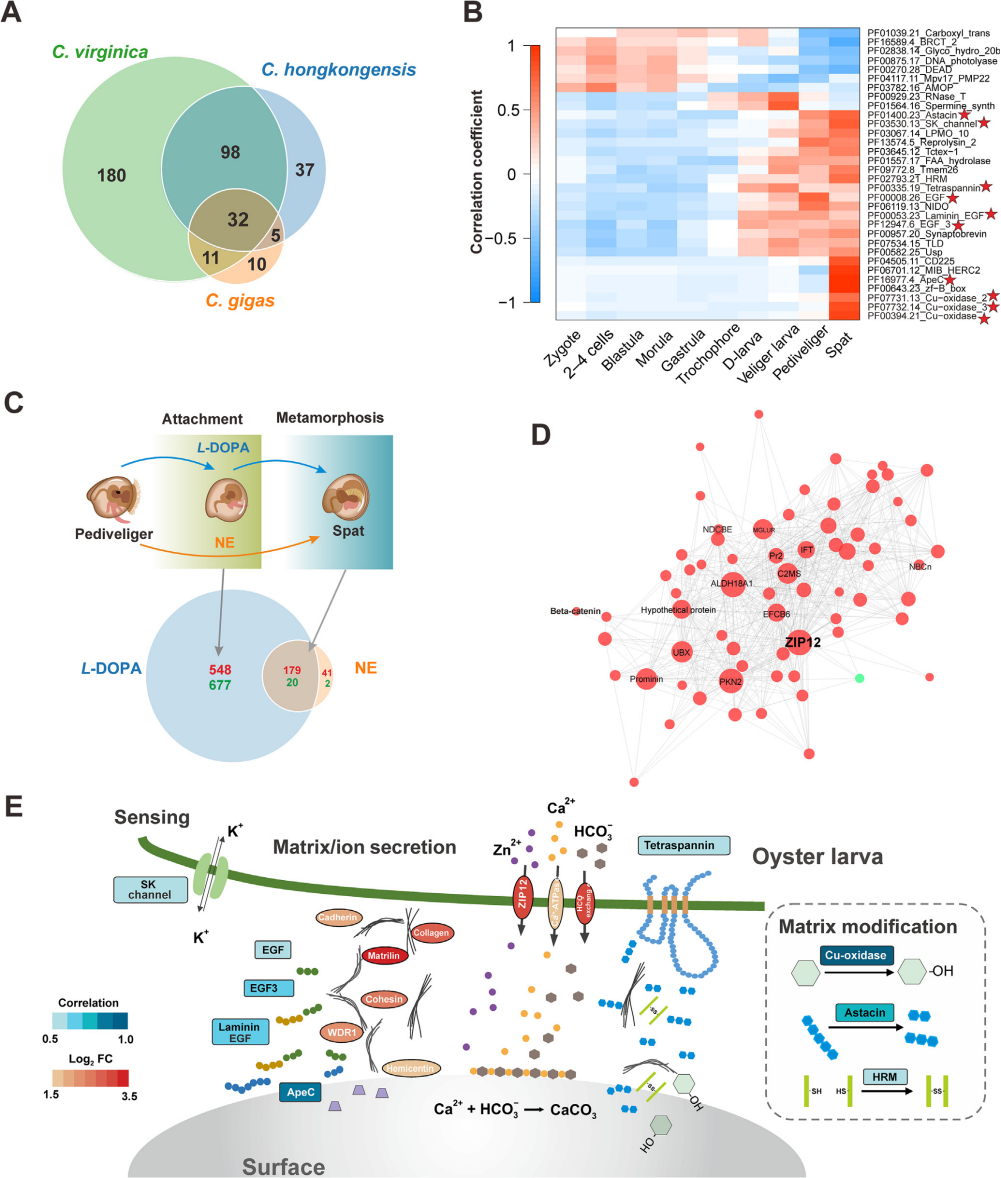
**Molecular basis of attachment initiation in Ostreoida oysters** **A.** Venn plot showing the commonly expanded gene families in three Ostreoida oyster species, *C. hongkongensis*, *C. virginica*, and *C. gigas*. A total of 32 core expanded gene families were identified. **B.** Heatmap illustrating the correlation between the expression levels of 32 core expanded gene families and the developmental stages of *C. hongkongensis* larvae. The high correlations between transcriptionally activated gene families and stages corresponding to attachment initiation in larvae are marked by red stars. **C.** Pharmacological responses of oyster pediveliger larvae during attachment initiation and metamorphosis. *L*-DOPA stimulates larval attachment and metamorphosis, while NE only induces metamorphosis without attachment. Venn plot shows the numbers of genes up-regulated (red) or down-regulated (green) by *L*-DOPA/NE treatment. The genes specifically altered by *L*-DOPA treatment may participate in attachment initiation. **D.** The coordinated gene network based on the zinc transporter ZIP12, which is a hub forming the highest degree of gene connections in WGCNA. Red and green dots indicate up-regulated and down-regulated genes, respectively. **E.** Schematic diagram conceptualizing the molecular basis for initiation of larval attachment in oysters. Square boxes indicate oyster-specific expanded gene families involved in larval attachment (*P* < 0.001). Filled color (blue) is scaled with correlation values at the spat stage. Ellipse boxes indicate *L*-DOPA specifically induced genes after *L*-DOPA treatment, which are filled in red scaled with values in log_2_ FC. *L*-DOPA, *L*-3,4-dihydroxyphenylalanine; NE, noepinephrine; WGCNA, weighted correlation network analysis; FC, fold change.

**Supplementary figure S7:**
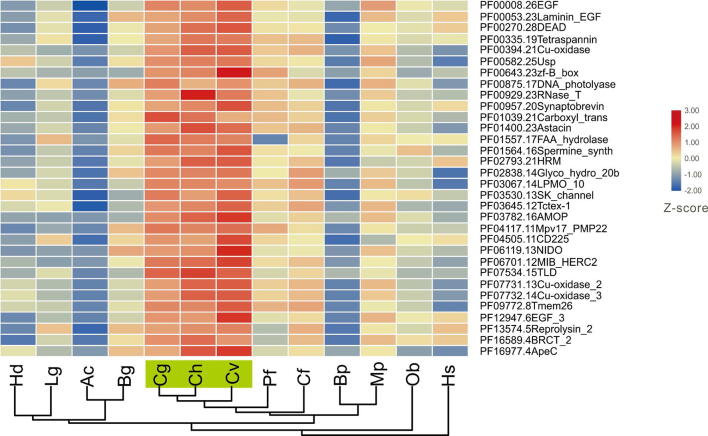
**Heatmap presents 32 common expanded gene families**. A heatmap was generated using TBtool software according to the gene number of each gene family. Evidently, these gene families are greatly expanded in three Ostreoida oysters under study, namely, *C. gigas, C. hongkongensis, and C. virginica. Haliotis discus hannai (Hd), Lottia gigantea (Lg), Aplysia californica (Ac), Biomphalaria glabrata (Bg), Crassostrea gigas (Cg), Crassostrea hongkongensis (Ch), Crassostrea virginica (Cv), Pinctada fucata (Pf), Chlamys farreri (Cf), Bathymodiolus platifrons (Bp), Modiolus philippinarum (Mp), Octopus bimaculoides (Ob), and Homo sapiens (Hs)*.

In place of byssal attachment, Ostreoida oysters adopt an ingeniously cost-effective way of sessile life, namely, cemented attachment [16]. Such adhesive mechanism is characterized by extraordinary mechanical strength and superior flexibility needed to resist powerful tidal scour and absorb surge energy [21]. Cemented attachment allows oysters to efficiently anchor and thrive in marine environments and ultimately supports the genesis and health of oyster reefs. Nevertheless, the molecular mechanisms underlying oyster adhesive production remain enigmatic. Taking into account that commented attachment is an innovation unique to Ostreoida oysters, we first ventured to investigate which gene families were expanded as a common event in three Ostreoida species. Our results showed that in *C. gigas*, *C. hongkongensis*, and *C. virginica*, there are 58, 172, and 321 species-specific expanded gene families, respectively, which can be further reduced to 32 core expanded gene families in Ostreoida oyster genomes (Figure 3A, Figure S7; Table S14).

**Supplementary figure S8:**
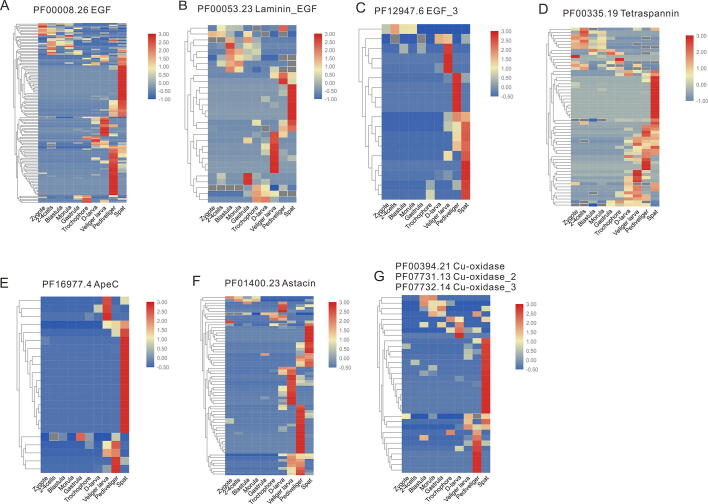
**Heatmaps of high-correlation gene families in oyster larval attachment**. Gene families of high correlation with larval attachment across developmental stages were analyzed, including *PF00008.26 Egf* (**A**), *PF00053.23 Laminin_Egf* (**B**), *PF12947.6 Egf 3* (**C**), *PF00335.19 Tetraspannin* (**D**), *PF16977.4 ApeC* (**E**), *PF01400.23 Astacin* (**F**), and *PF00394.21, PF07731.13*, and *PF07732.4 Cu-oxidase* (**G**). Changes in gene expression levels during different developmental stages are schematically depicted. Heatmaps were generated using TBtool software. Developmental stages are abbreviated as zygote, 2−4 cells, blastula, morula, gastrula, trochophore, D-larve, veliger larva, pediveliger, and spat.

To elucidate how the expansion of these core gene families facilitates cemented attachment, we analyzed the correlations between their expression levels and specific developmental stages (Figure 3B; Table S15). Developmentally, attachment is an intricate secretion process involving a broad spectrum of chemical reactions and proteins, notably extracellular enzymes or matrices [22]. It was thus unsurprising that a small-conductance calcium-activated potassium (SK) channel gene family and nine extracellular gene families were identified to be involved in this process, which showed high correlations in the pediveliger and spat stages corresponding to the attachment initiation in larvae (Figure 3B). SK channels are widely expressed calcium-activated potassium channels in neurons, playing crucial roles in regulating dendritic excitability and synaptic plasticity [23]. Interestingly, increased expression of expanded SK channels may aid free-swimming larvae in sensing external environments in search of an appropriate attachment site. On the other hand, the function of extracellular gene families is strictly related to key processes of shell attachment, including matrix secretion [epidermal growth factor (*Egf*), *Egf3*, lamin *Egf*, and *ApeC*], processing of matrix modification (*Cu-oxidase*, *Cu-oxidase2*, *Cu-oxidase3*, and *Astacin*), among others (Figure 3B). Indeed, many adhesive proteins contain specific protein-binding domains [24], such as EGF-like domains in the slug mucus proteins (*e.g.*, SM40 and SM85) [25] and sea star footprint proteins (*e.g.*, SF1) [26], raising the possibility that EGF family expansion in *C. hongkongensis* is functionally linked to the cemented attachment. Additionally, physico-chemical properties of many adhesive proteins arise in part from post-translational modifications, which ultimately support their adhesive functions [24]. Protein oxidation in marine bio-adhesives indeed contributes to enhanced crosslinking between shell disks and substrates during attachment [27]. A notable gene expansion in the copper oxidase family is likely to contribute to the stabilization of extracellular matrixes in the form of crosslinking between the oyster shell and external substrates. Copper-based enzyme lysyl oxidase is known to be essential for crosslinking and strengthening fibers in animal connective tissues via collagen oxidation [28]. Concomitantly, copper ion, as part of oxidative enzymes, is a mandatory cofactor for oxidase activity, which creates crosslinking sites from common amino acids, to enhance the cemented attachment [29], [30]. Further transcriptomic analysis showed that the nine extracellular gene families were significantly up-regulated during the larvae-spat transformation of embryo development stages (Figure S8), confirming their functional importance in attachment formation.

### *L*-3,4-dihydroxyphenylalanine-induced attachment

**Supplementary figure 9:**
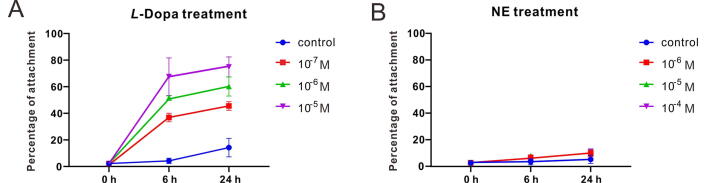
**Effects of *L*-3,4-dihydroxyphenylalanine and noradrenaline treatments on oyster larval attachment**. *L*-DOPA induced normal attachment and metamorphosis, whereas NE induced metamorphosis without attachment. Low-concentration (10^-6^ M) *L*-DOPA treatment caused an increase in adhesion rate, while medium- (5×10^-6^ M) and high-concentration (10^-5^ M) treatments caused an increase in adhesion rate within a short time (3 h). NE induced morphological changes in larvae, and treatment at 3 h and 6 h significantly induced metamorphosis. There were no significant induction effects after 9 h treatment. We also determined the survival rate of oyster larvae after 30 h of drug treatment. Results show that all experimental concentrations used in drug treatment did not affect larval survival rate compared to that of the control group. *L*-DOPA, *L*-3, 4-dihydroxyphenylalanine; NE, noradrenaline.

During larvae-spat transformation, embryonic oysters execute an intrinsic program of developmental changes, in which cemented attachment is tightly coupled to metamorphosis [31]. In this context, we set out to distinguish molecular determinants of cemented attachment from that of metamorphosis at the pediveliger stage by means of two pharmacologic agents: *L*-3,4-dihydroxyphenylalanine (*L*-DOPA) and norepinephrine (NE). The former simultaneously promotes normal attachment and metamorphosis, whereas the latter induces metamorphosis only but not attachment (Figure 3C, Figure S9) [31]. Based on this, a gene whose expression was up-/down-regulated by *L*-DOPA treatment rather than NE treatment was hypothesized to be a driver for attachment initiation in *C. hongkongensis*. We accordingly scrutinized 24 transcriptomes following pharmacological challenges at two time points within the temporal span of oyster attachment. Our results show that the expression of 1225 genes was specifically altered by the treatment of *L*-DOPA rather than NE (Figure 3C), confirming the essential role of *L*-DOPA as an attachment signal.

**Supplementary figure S10:**
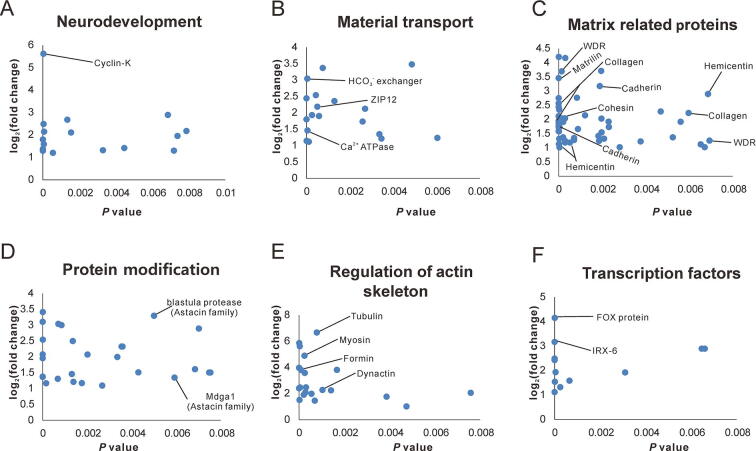
**Transcriptomic analysis post-treatment**. Transcriptome sequencing was conducted to assess the specific expression of 1424 genes after *L*-DOPA treatment, involved in neurodevelopment (**A**), material transport (**B**), matrix-related proteins (**C**), protein modification (**D**), regulation of actin skeleton (**E**), and transcription factors (**F**). Some important genes are marked in the figure. The *x*-axis represents *P* values, and the *y*-axis represents fold change.

Remarkably, the expression levels of several genes encoding neurotransmitter receptors (including metabotropic glutamate receptor and neuropeptide Y receptor) were significantly increased, consistent with the assumption that neuromuscular coordination is essential for guiding embryos to settle in suitable niches and initiate attachment (Figure S10) [32]. Moreover, genes encoding metal ion channels or binding proteins were significantly enriched, with notable examples like organic cation transporters, transient receptor potential cation channels (*e.g.*, ZIP12), and voltage-dependent calcium channels (*e.g.*, Ca^2+^-ATPase), which is intuitively consistent with the well-documented stimulatory roles of selective cations in oyster larval settling. To highlight, potassium voltage-gated channel activity has been proven to be vital for oyster larval attachment, since its inhibitor tetraethyl ammonium can effectively block this developmental process [33]. Typically, attachment initiates in oyster larvae with the aid of fibrous adhesive proteins and other bioorganic substances, including mucopolysaccharides and phospholipids [2]. As a consequence, extensive extracellular matrix and adhesion proteins including collagen, cadherin, fibrocystin, and hemicentin would increase in response to *L*-DOPA simulation, presumably paving the way for larval attachment [34].

**Supplementary figure S11:**
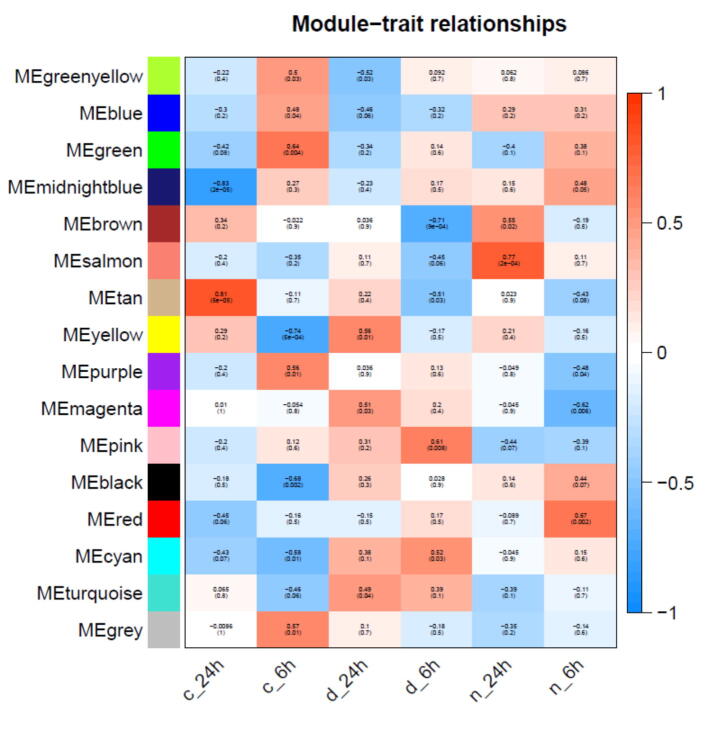
**Weighted correlation network analysis on module-trait relationships**. The top 10,000 differentially expressed genes exhibiting transcriptional changes in response to *L*-DOPA treatment were selected for WGCNA, wherein MEpink modules showed a high correlation with *L*-DOPA induced attachment.

**Supplementary figure S12:**
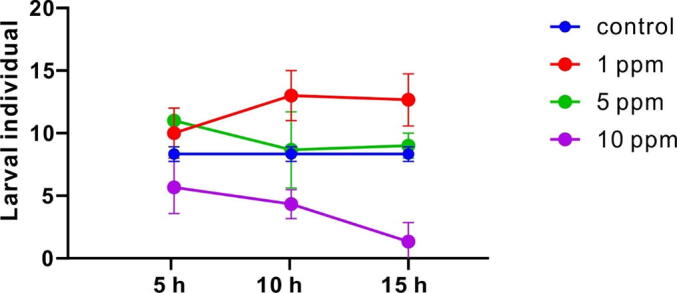
Low-dose zinc induces attachment. Oyster larvae were treated with different concentrations of ZnCl2 (1 ppm, 5 ppm, and 10 ppm) for 5 h, 10 h, and 15 h. The control groups were treated with equal volumes of seawater. Each group contained three replicates, and independent experiments were performed three times. Error bars represent means ± SEM (*n*=3). Ppm, parts per million.

To search out the crucial molecular determinants governing this process, we performed weighted correlation network analysis (WGCNA) to construct a potential connected gene network functionally associated with *L*-DOPA-induced attachment, wherein 15 modules were subsequently identified (Figure S11). Among them, the MEpink module was most correlated with *L*-DOPA-induced attachment (*P* < 0.01) and contained 139 genes (topological overlap > 0.3). Intriguingly, within this module, a hub forming the most connections in the network was found to be the zinc transporter ZIP12 (Figure 3D), which is a pivotal regulator of zinc flux. As a cofactor essential to a wide spectrum of proteins such as matrix metalloproteinases, zinc plays vital regulatory roles in enzymatic catalysis and macromolecular stability [35]. A high abundance of zinc is also a salient feature in aragonite- or calcite-rich shells in certain mollusks [36]. Meanwhile, among the gene families that were specifically expanded in Ostreoida oysters, *Astacin* encodes a cell-secreted or plasma membrane-associated protease that possesses zinc-binding activity and takes part in proteolytic processing of extracellular proteins [37]. Its expression was markedly elevated both during larvae-spat transformation and larval response to *L*-DOPA treatment (Figures S8G and S10D). Predictably, chelation of zinc potently retarded oyster larval attachment (Figure S12), providing additional hints that the initial creation of matrix structures requires zinc and associated protein activities for cement attachment. Accordingly, based on genomic results on extracellular gene family expansion and transcriptomic profiles at the attachment stage, we conceived a conceptual model to delineate the mechanistic determinants and processes working in the cement attachment strategy of oyster larvae (Figure 3E). We postulate that attachment formation apparently results from intricate coordination of at least three types of fundamental activities, namely, larval sensing of habitable surfaces, matrix/ion secretion, and matrix modification to mobilize adhesive processes.

### Asymmetry in left-right shell formation

**Figure 4:**
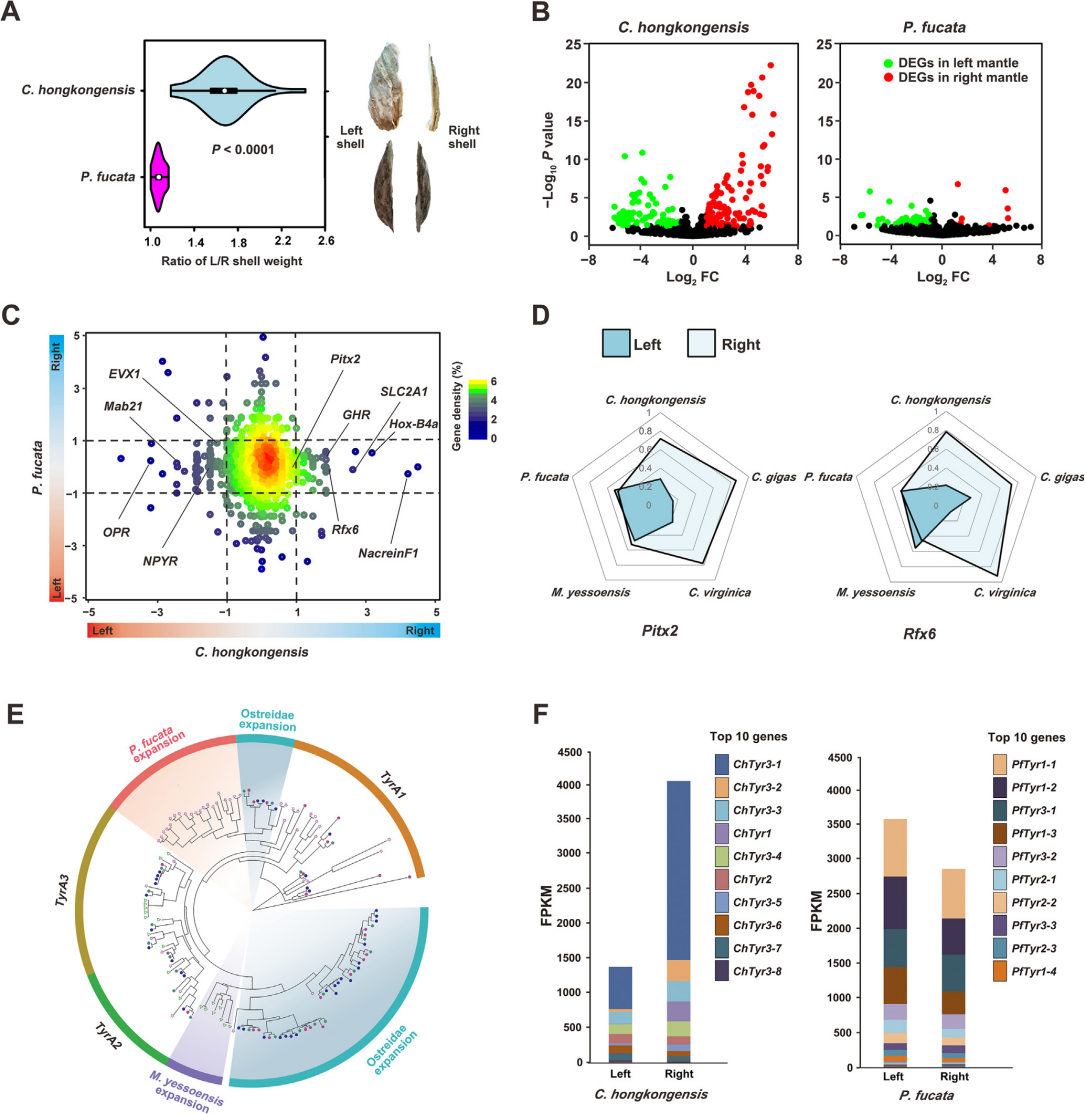
**Left-right asymmetry of shell formation in Ostreoida oysters** **A.** Comparison of the ratio of L/R shell weight and morphology between *C. hongkongensis* and *P. fucata*. **B.** Volcano plot showing the left- and right-mantle DEGs, which are filtered by |log_2_ FC| ≥1 with *P* < 0.05. **C.** Expression profile of paired orthologues in L/R mantles of *C. hongkongensis* and *P. fucata*. A total of 10,491 orthologues were paired and only a few asymmetry-related orthologues were specifically expressed in *C. hongkongensis*. The *x*- and *y*-axes indicate log_2_ FC of expression ratio in R/L mantles of *C. hongkongensis* and *P. fucata*, respectively. **D.** Expression patterns of two pivotal transcription factor genes in left and right mantles across five bivalves. **E.** Dendrogram of known *Tyr* genes from five mollusks constructed by maximum likelihood method. Bivalve and molluscan *TyrA* orthologous groups are indicated by curvatures and annotated as *A1*–*A3*. Specific *Tyr* orthologous groups are marked with colored backgrounds and annotated with a species’ name. Species are represented with different shapes: triangle, *M*. *yessoensis*; circle, Ostreidae; pentagon, *P*. *fucata*. **F.** Expression patterns of *Tyr* gene families in left and right mantles of two bivalve species, as determined by FPKM. Total FPKM of different types of orthologous genes is displayed in cumulative histograms. Different members of *Tyr* gene families are presented with different colors. L/R, left-right; DEG, differentially expressed gene; *Tyr*, tyrosinase; FPKM, fragments per kilobase per million.

**Supplementary figure S13:**
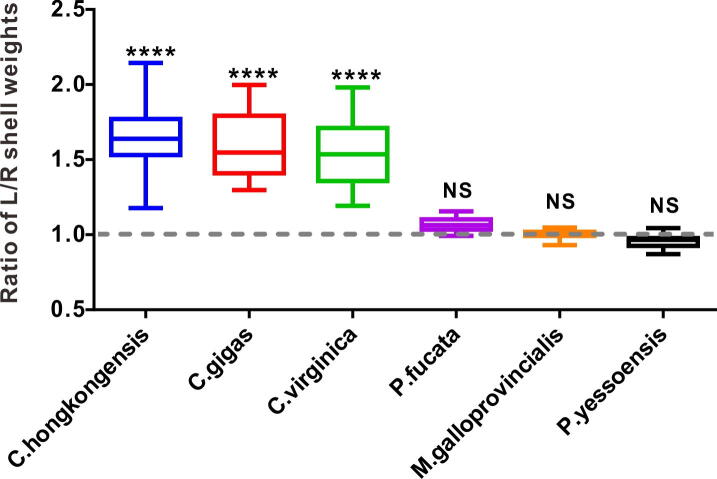
**Ratio of left/right shell weights in different bivalve species**. Weights of the left and right shells of several representative bivalves were measured, and their statistical results are as shown in the figure. Forty individuals per species were used for the assessment. The left and right shell weights of six bivalves are represented in a box plot. The dotted line indicates a weight ratio of 1. ****, *P* < 0.0001. NS, no significance.

**Supplementary figure S14:**
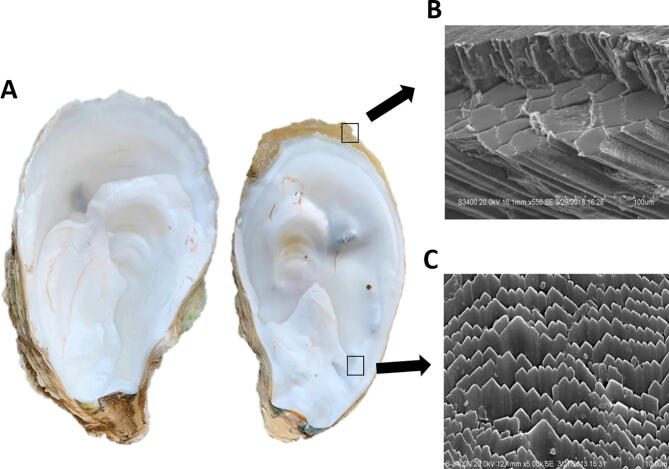
Scanning electron micrographs of the left and right shells of the Hong Kong oyster. Oyster shells consist of a prismatic layer and a foliate layer spanning from outside to inside and are chemically composed of CaCO3. **A**. The left and right shell of the Hong Kong oyster. **B**. The top side of the right oyster is the prismatic layer. **C**. The inner side of the right shell is the foliate layer. SEM magnified the object 550 times (B) and 5000 times (C).

Symmetry is an elegant guiding principle for the implementation of body plans [38]. Across the Bivalvia class, the majority of bivalves display perfect or near-perfect conformity to bilaterally symmetrical shells [8], [39]. In contrast, Ostreoida oysters may appear unorthodox in adopting morphological asymmetry in their shell formation due to functional differentiation of the L/R shells (Figure S13). The left shell is visibly much thicker and more convex than its right counterpart, which is apt for attaching to rocky surfaces or neighboring oysters within a reef community. On the other hand, the right shell is capable of physical displacement and hermetic lockdown to regulate water intake and ward off predation (Figure 4A). Moreover, structural variance in shell asymmetry is also amply reflected by a greater proportion of prismatic layers in the right shell (Figure S14), which are responsible for controlling the initiation of calcite crystal formation and growth [40]. Although asymmetry of body forms has been traditionally stereotyped as defects that may jeopardize the survival of an organism [41], the example of Ostreoida oysters clearly defies this rule. We reason that such an intriguing differentiation of asymmetrical shells could confer unexpected benefits such as improved population fitness in an otherwise intrinsically harsh coastal environment. With the advent of the left shell and its versatile attachment machinery, oysters can easily economize resources or secure their foothold on rocks or peers’ shells within an oyster reef via cemented attachment [42]. This strategy permits oysters to lower their thresholds for founding and expanding productive colonies in demanding physical habitats, literally through the stacking of individuals at high densities, without sacrificing resistance to environmental challenges such as tidal turbulences.

To further elucidate the molecular basis of left-right asymmetry, comparative transcriptomic analysis was carried out to quantify the gene expression profiles in the L/R mantles of *C. hongkongensis* and pearl oyster, which are the key organ controlling shell formation [43]. As expected, 188 asymmetry-related differentially expressed genes (DEGs) were identified in the L/R mantles of *C. hongkongensis*, whereas only 53 asymmetry-related DEGs were found in the L/R mantles of the pearl oyster (Figure 4B), which reflects a radical genetic divergence underpinning shell asymmetry. Next, to test the hypothesis that lineage-specific divergence of orthologues contributes to symmetry breakage, 10,491 of the orthologues were paired between the two species. Our results indicate that a few but crucial asymmetry-related orthologues are specifically expressed *C. hongkongensis* (Figure 4C), including homeobox gene paired-like homeodomain transcription factor (*Pitx2*), homeobox B4a (*Hox-B4a*), and regulatory factor *X6* (*Rfx6*). Notably, *Pitx2* is a central regulator orchestrating the Nodal cascade, which is responsible not only for directing L/R axis formation in mammals [44], but also for shell coiling and L/R asymmetry in some mollusks such as the snail [45]. Another gene of interest is *Rfx6*, recognized for its fundamental importance in guiding pancreatic islet development and insulin production in mammals [46]. While the insulin-related peptide gene is known for being a critical driver of oyster growth [47], this new evidence suggests novel roles of Rfx6–insulin signaling in maintaining shell asymmetry in oysters. As predicted, asymmetry-related expression of *Pitx2* and *Rfx6* in L/R mantles was confirmed by real-time qPCR in three Ostreoida lineages with asymmetrical shells, whereas such gene expression patterns were absent in three symmetrical bivalves, pearl oyster, scallop, and mussel (Figure 4D).

**Supplementary figure S15:**
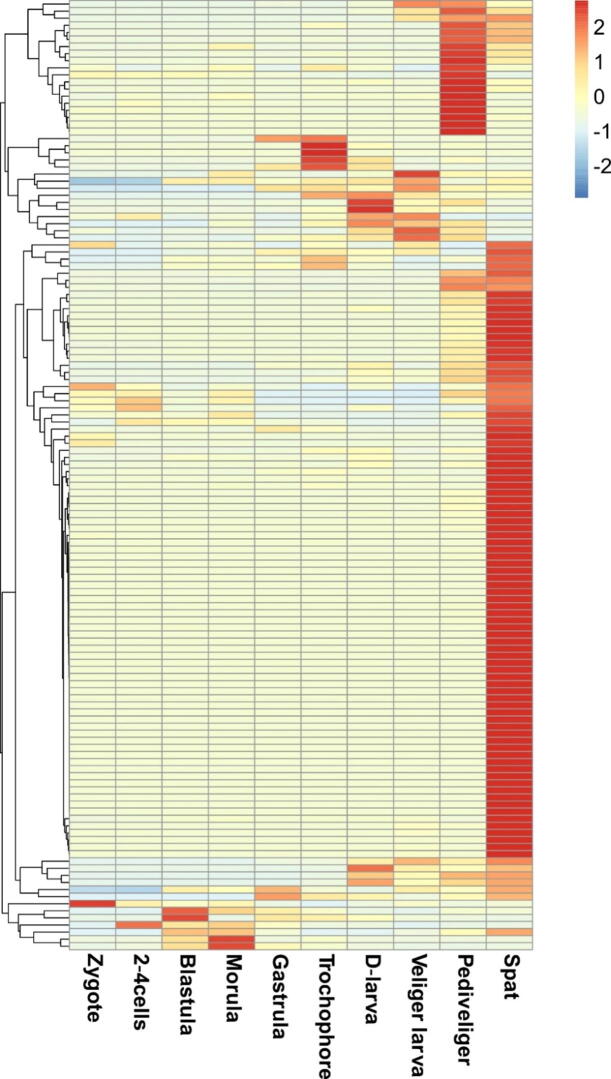
**Shell asymmetry-related gene expression profiles seen during the developmental stages**. To study the molecular mechanisms underlying asymmetric growth of oyster shells in the Hong Kong oyster, expression patterns of 134 differentially expressed genes were analyzed during developmental stages. Developmental stages are abbreviated as zygote, 2−4 cells, blastula, morula, gastrula, trochophore, D-larve, veliger larva, pediveliger, and spat.

However, it should be noted that the majority of asymmetry-related genes in *C. hongkongensis* are not orthologous to those in the pearl oyster. For example, tyrosinase (*Tyr*) genes are one of the key gene families involved in steering shell formation and pigmentation by means of oxidation and crosslinking of o-diphenols [48]. Phylogenetic analysis revealed that more than half of *Tyr* genes (55%) clustered in several lineage-restricted clades, suggesting the rapid and independent expansion of this gene family in bivalves (Figure 4E). Remarkably, several high-abundance members of the *Tyr* family seemed to be strongly associated with L/R asymmetry and were expressed preferentially in the right mantles of *C. hongkongensis*, whereas no obvious variance was noted between left and right mantles in the pearl oyster (Figure 4F). Therefore, it seems logical to infer that rapid expansion and divergent expression of the *Tyr* gene family contribute importantly to the emergence and neofunctionalization of asymmetrical shell formation in Ostreoida lineages. Lastly, we found that 71.1% of these genes started expression at the spat stage (Figure S15), implying that a complete asymmetrical pattern becomes established in the juveniles only after metamorphosis.

## Conclusion

Ostreoida oysters have evolved remarkable innovations for streamlining their body plans, which are enabled by novel cemented attachment and allied gene machinery diverging from L/R symmetry. These evolutionary breakthroughs poise oysters as highly successful reef builders and ecological guardians integral to marine ecosystems spanning the globe. To reveal the genomic changes driving these evolutionary innovations, we sequenced the complete genome of *C. hongkongensis*, obtained active transcriptomic data developmentally critical to the attachment window, and made comparisons with other bivalve genomes. The *Antp* gene of the *Hox* cluster, found to be lost in Ostreoida oysters, is evidently a pivotal regulator of byssal secretion and expression of byssal proteins in *P. fucata*, and potentially a critical gene governing the radical switch from byssal to the cemented attachment. Furthermore, extensive extracellular gene families were expanded in the Ostreoida lineages specifically, presumably contributing to the operationalization of cemented attachment. Ion-binding genes were significantly enriched in *L*-DOPA-induced attachment in oysters, with zinc-binding genes being a prominent network that coordinates extracellular matrix modification and initiates adhesion. Moreover, Ostreoida divergence from shell symmetry is probably under the joint control of a suite of transcriptionally identified asymmetry-related DEGs in the L/R mantles, notably the transcription factor genes *Pitx2* and *Rfx6*, as well as the expanded lineage-specific family encoding tyrosinases. Thus, on the basis of genomic determinants and coordinated gene networks as revealed in this study, we have advanced a detailed picture of how shell asymmetry is switched on and driven in bivalves such as Ostreoida oysters. In order to provide insights into bivalve biology and disease in contexts of climate change or biological conservation, further investigation on the attachment-governing genes may be warranted.

## Materials and methods

### Illumina sequencing

Genomic DNA was extracted by using DNeasy Blood & Tissue Kit (Catalog No. 69582, Qiagen, Hilden, Germany) from a two-year-old single individual of *C. hongkongensis*. Two types of pair-end libraries (220 bp and 500 bp) and six types of long-insert mate-pair libraries (3 kb, 4 kb, 5 kb, 8 kb, 10 kb, and 15 kb) were constructed by using Illumina’s paired-end and mate-end kits, according to the manufacturer’s instructions. Libraries were sequenced on an Illumina Hiseq 2500 platform. For raw reads, sequencing adaptors were removed. Contaminated reads (such as chloroplast, mitochondrial, bacterial, and viral sequences) were screened by alignment in accordance with an NCBI-NR database by using Burrows-Wheeler alignment (BWA) v0.7.13 with default parameters. FastUniq v1.1 was used to remove duplicated read pairs. Low-quality reads were filtered out, according to the following criteria: 1) reads with ≥ 10% unidentified nucleotides (N); 2) reads with > 10 nucleotides aligned to an adapter, allowing ≤ 10% mismatches; 3) reads with > 50% bases with Phred quality < 5.

### PacBio sequencing

Genomic DNA was sheared by a g-TUBE device (Catalog No. 520079, Covaris, MA) with 20 kb settings. Sheared DNA was then purified and concentrated with AMPure XP beads (Catalog No. 10136224, Beckman Coulter, CA) and further used for single-molecule real-time (SMRT) bell preparation according to the manufacturer’s protocol (Pacific Biosciences, CA) and 20 kb template preparation by using BluePippin size selection (Sage Science). Size-selected and isolated SMRT bell fractions were purified with AMPure XP beads. Finally, these purified SMRT bells were used for primer and polymerase (P6) binding, according to the manufacturer’s binding calculator (Pacific Biosciences). Single-molecule sequencing was performed on a PacBio RS-II platform with C4 chemistry. Only PacBio subreads ≥ 500 bp were included for performing oyster genome assembly.

### Genome size estimation

About 34 Gb (52×) corrected Illumina reads from the 180-bp and 500-bp libraries were selected to perform genome size estimation. The oyster genome size was estimated based on the formula: genome size = *k*-mer number/peak depth.

### ***De novo*** genome assembly of Illumina data

Clean Illumina reads were assembled *de novo* into longer contigs by using ALLPATH-LG [49] with default parameters. Adjacent contigs were linked to scaffolds by leveraging mate-pair information with SSPACE v2.3 [50], while gaps were filled by using GapCloser v1.12 [50] implemented in a SOAPdenovo2 package [51].

### ***De novo*** genome assembly of PacBio data

#### Canu+LoRDEC+WTDBG

We used an error correction module of Canu v1.5 [52] to select longer subreads with the settings ‘genomeSize = 3,500,000,000’ and ‘corOutCoverage = 80’, detected raw subreads overlapping through a highly sensitive overlapper MHAP v2.12 (‘corMhapSensitivity = low/normal/high’), and completed an error correction through a falcon_sense method (‘correctedErrorRate = 0.025’). Subsequently, output subreads of Canu were further corrected by LoRDEC v0.6 [53] with the parameters ‘-k 19 -s 3’. Based on these two rounds of error corrections, we generated a draft assembly by using WTDBG 1.1.006 (https://github.com/ruanjue/wtdbg) with the command ‘wtdbg -i pbreads.fasta -t 64 -H -k 21 -S 1.02 -e 3 -o wtdbg’.

#### Hybrid genome assembly

Contigs produced by ALLPATH-LG were optimized with the aid of contigs of PacBio assembly by using quickmerge with the parameters ‘-hco 5.0 -c 1.5 -l 100,000 -ml 5000’. Optimized contigs were linked to scaffolds by leveraging Illumina mate-pair information by using SSPACE, and gaps were filled by using PBjelly v2.

#### Evaluation of oyster assembly

To estimate the genome quality, we first mapped Illumina reads to the oyster assembly by using BWA tool. Next, the completeness of genomes was verified by mapping 248 highly conserved eukaryotic genes and 908 BUSCOs in metazoa to the genomes by using CEGMA v2.5 [54] and BUSCO v3.0.2b [55], respectively.

### Hi-C sequencing and assembly

#### Sequencing

According to the Hi-C procedure [56], nuclear DNA from muscles of oyster individuals was crosslinked, and then excised with a restriction enzyme, leaving pairs of distally located but physically intercalated DNA molecules attached to one another. The sticky ends of these digested fragments were biotinylated, which were then ligated to each other to form chimeric circles. Biotinylated circles, as chimeras of physically associated DNA molecules from the original crosslinking, were enriched, sheared, and sequenced [57]. After adaptor removal and filtering out low-quality reads, Hi-C reads were aligned to our assembled genome to evaluate ratio of mapped reads, distribution of insert fragments, sequencing coverage, and number of valid interaction pairs. Uniquely mapped reads spanning two digested fragments that are distally located but physically associated DNA molecules are defined as valid interaction pairs.

#### Assembly

Scaffolds of PacBio and Illumina assemblies were reduced to fragments with a length of 300 kb, which were then re-assembled by using the LACHESIS software [57] based on Hi-C data. Regions that failed to be restored to the original assembly or contained an average Hi-C data coverage of less than 0.5% were considered assembly errors and were broken into smaller scaffolds. Consistency in the assembly of Hi-C data based pseudo-chromosomes was assessed by comparisons with a genetic map for *C*. *gigas* [58] by using the software of ALLMAPS.

### Genome annotation

#### Repetitive sequence prediction

Repeat composition of the assemblies was estimated by building a repeat library employing the *de novo* prediction programs LTR-FINDER, MITE-Hunter, RepeatScout, and PILER-DF. The database was classified by using PASTEClassifier [59] and then combined with the Repbase database [60] to create a final repeat library. Repeat sequences in the oyster genome were identified and classified by using the RepeatMasker program [61]. The classification criterion for long termimal repeat (LTR) family was defined as that 5′-LTR sequences of the same family would share at least 80% identity over at least 80% of their lengths.

#### Protein-coding gene prediction

Protein-coding genes were predicted based on *ab initio* prediction and protein homology-based approaches. The algorithms Genscan [62], Augustus [63], GlimmerHMM [64], GeneID [65], and SNAP [66] were used for *ab initio* prediction. Alignment of homologous peptides from *C. gigas*, *C. virginica*, *Lottia gigantea*, and *Danio rerio* to our assemblies was performed to identify homologous genes with the aid of GeMoMa [67]. Consensus gene models were generated by integrating the *ab initio* predictions and protein alignments using EVidenceModeler (EVM) [68].

#### Functional annotation of protein-coding genes

Annotation of the predicted genes was performed by blasting their sequences against a number of nucleotide and protein sequence databases, including COG, KEGG, NCBI-NR, and Swiss-Prot, with an E-value cutoff of 1E−5. Gene Ontology (GO) for each gene was assigned by using Blast2GO [69] based on NCBI databases.

### Evolution of oysters

Protein sequences of *Haliotis discus hannai* [70], *L*. *gigantea* (GCF_000327385.1), *Aplysia californica* (GCF_000002075.1), *Biomphalaria glabrata* (GCF_000457365.1), *C*. *gigas* (GCF_000297895.1), *C*. *virginica* (GCF_002022765.2), *P*. *fucata* (https://marinegenomics.oist.jp), *C*. *farreri* (CfBase), *B*. *platifrons* (GCA_002080005.1), *M*. *philippinarum* (GCA_002080025.1), *Octopus bimaculoides* (GCF_001194135.1), and *Homo sapiens* (GCF_000001405.26) were retrieved for analysis. Proteomes of the aforementioned 12 species and that of *C. hongkongensis*, comprising a total of 295,905 protein sequences, were clustered into 38,939 orthologous groups by using OrthoMCL v3.1 [71] based on an all-to-all BLASTP strategy with an E-value of 1E−5 and by using markov chain clustering (MCL) algorithms with default inflation parameters (1.5). Based on clustering results, *C. hongkongensis*-specific gene families were determined and annotated. To infer phylogenetic relationships, we extracted 387 single-copy gene families from all 13 species to perform multiple alignments of proteins for each family with MUSCLE v3.8.31 [72]. All of the alignments were combined into one supergene to construct a phylogenetic tree by using RAxML v8.2.12 [73] with 1000 rapid bootstrap analyses, followed by a search of the best-scoring Maximum likelihood tree in a single run. Finally, divergence time was estimated by using MCMCTree from the PAML package [74] in conjunction with a molecular clock model. Several reference-calibrated time points obtained from TimeTree database (http://timetree.org/) were used to date divergence time of interest. Expansion and contraction of OrthoMCL-derived homolog clusters were determined by CAFÉ v2.1 [75] on the basis of changes in gene family size with respect to phylogeny and species divergence time. In addition, we obtained domain-based expanded gene families of three *Crassostrea* species, according to previous work by Albertin and colleagues [76].

### Syntenic analysis

All-to-all BLASTP analyses of protein sequences were performed between *C. hongkongensis*, *C. gigas*, *C. virginica*, and *P. fucata* with an E-value threshold set at 1E−5. Syntenic regions within and between species were identified by using MCScan based on BLASTP results. A syntenic region was considered valid if it contained a minimum of 10 collinear genes and a maximum of 25 gaps (genes) between two adjacent collinear genes.

### *Hox* gene analysis

Structures of *Hox* genes in oysters were determined by using the GeMoMa v1.4.2 software [77] with default parameters based on available homeobox gene models. Predictions were handled by applying a GeMoMa annotation filter (GAF) with default parameters except for the evidence percentage filter (e = 0.1). These were then manually verified to achieve a single high-confidence transcript prediction per locus. Exact annotations of each homeobox gene were completed with the aid of phylogenetic relationships.

### Transcriptomic analysis

Embryos at different developmental stages during oyster embryogenesis, including zygote, 2–4 cells, blastula, morula, gastrula, trochophore, D-larva, veliger, pediveliger, and spat, were collected for RNA isolation. Similarly, RNA extraction was done with various tissues, including hemocytes, muscles, gill, labial palp, hepatopancreas, gonads, and mantles. To compare asymmetry-related gene expression in the mantles of *C. hongkongensis* and *P. fucata*, their L/R mantles were collected. For both left and right mantles, unilateral tissues from five individuals were pooled as one sample, and each of the L/R mantle groups contained at least three replicates. Total RNA was isolated by using the Trizol reagent (Catalog No. 15596026, ThermoFisher Scientific, Waltham, MA), followed by treatment with RNase-free DNase I (Catalog No. M6101, Promega, WI), according to the manufacturers’ instructions. RNA quality was then checked by using an Agilent 2100 Bioanalyzer. Illumina RNA-seq libraries were prepared and sequenced in a HiSeq 2500 system by a PE150 strategy following the manufacturer’s instructions (Illumina, CA). After trimming raw reads based on quality scores from the quality trimming program Btrim, clean reads were aligned to the oyster assembly genome by using TopHat v2.1.1 [78] and then assembled by using Cufflinks v2.1.1 [79]. Differential expression of genes in various tissues was evaluated by using Cuffdiff [79].

### WGCNA and co-expression network analysis

WGCNA [80] was applied to construct a weighted gene co-expression network for genes having a high correlation with cemented attachment. The top 10,000 differential genes exhibiting transcriptional changes in response to *L*-DOPA treatment were selected for WGCNA, wherein the modules showed a high correlation with cemented attachment. We estimated the weight for each pair of genes forming intersections within these modules and analyzed DEGs relevant to cemented attachment by using DESeq2. Cytoscape was used to delineate the co-expression network of significant gene pairs with weight > 0.3.

### Byssal regeneration

Functional relationships between *Antp* mRNA expression levels and phenotypic traits of byssal threads in adult pearl oysters (*P*. *fucata*) were explored. Briefly, 50–100 pearl oysters (2 years old) were collected and maintained in aerated laboratory tanks. Byssal mass comprising the byssal stem and existing old threads of pearl oysters were excised. Then, individual pearl oysters were placed in beakers (one oyster per beaker) to allow identification of subsequent regrowth of nascent thread mass. Particular care was taken in removing old threads and attachment discs from the shells. Preliminary experiments indicate that removal of the threads did not affect subsequent thread formation. Byssal thread formation was estimated as the number of threads per oyster observed 24 h later.

Subsequently, the corresponding byssal gland of each pearl oyster was collected for RNA extraction by using TRIzol reagent, according to the manufacturer’s instructions. Purified RNA samples were diluted to 1 µg/µl and pooled to perform cDNA synthesis by utilizing PrimerScript 1st Strand cDNA Synthesis Kit (Catalog No. 6110A, Takara, Japan), following the manufacturer’s protocol. Real-time qPCR analysis was performed to determine *Antp* mRNA expression with gene-specific primers (Table S16).

### Pharmacological treatment

Chemical compounds were obtained from Sigma-Aldrich, unless otherwise specified. Working solutions were freshly prepared in deionized (DI) water approximately 1 h before *in vivo* experiments, which were conducted in large beakers to allow observation of oyster attachment and metamorphosis. Groups of oyster larvae at the pediveliger stage were placed in three beakers containing 50 ml seawater (at a density of 20 larvae/ml). There were three groups in total: an unstimulated control, an *L*-DOPA-treated group, and a NE-treated group. Oyster larvae were challenged with different concentrations of NE (1 × 10^−4^, 1 × 10^−5^, 1 × 10^−6^ M) or *L*-DOPA (1 × 10^−5^, 1 × 10^−6^, 1 × 10^−7^ M). Previous studies have shown that this concentration range is sufficiently potent for inducing a larval response [81].

Then, oyster larvae were collected 6 h and 24 h after treatment for RNA-seq and transcriptomic analyses to determine any temporally driven differences between the *L*-DOPA-treated group (1 × 10^−5^ M) and the unstimulated control. By a similar design, oyster larvae were exposed to NE (1 × 10^−5^ M) for 6 h and 24 h, and their transcriptomic profiles were examined in relation to oyster metamorphosis.

## Data availability

The raw sequence data reported in this study have been deposited in the Genome Sequence Archive [82] at the National Genomics Data Center (NGDC), Beijing Institute of Gemonics (BIG), Chinese Academy of Sciences (CAS) / China National Center for Bioinformation (CNCB) (GSA: CRA004099), and are publicly accessible at https://ngdc.cncb.ac.cn/gsa. The whole-genome sequence data reported in this study have been deposited in the Genome Warehouse [83] at the NGDC, BIG, CAS / CNCB (GWH: GWHBAZL00000000), and are publicly accessible at https://ngdc.cncb.ac.cn/gwh. The *C. hongkongensis* genome studied in this Hong Kong oyster genome project has also been deposited in the BioProject database at NCBI (BioProject: PRJNA592306), and are publicly accessible at https://www.ncbi.nlm.nih.gov/bioproject/PRJNA592306. Hi-C data have been deposited in the BioSample database at NCBI (BioSample: SAMN13420518), and are publicly accessible at https://www.ncbi.nlm.nih.gov/sra/SRR10583824. RNA-seq data of various transcriptomes have been deposited in the BioProject database at NCBI (BioProject: PRJNA588628), and are publicly accessible at https://www.ncbi.nlm.nih.gov/bioproject/PRJNA588628.

## CRediT author statement

**Yang Zhang:** Conceptualization, Methodology, Validation, Investigation, Data curation, Writing - original draft, Writing - review & editing, Visualization, Supervision, Project administration, Funding acquisition. **Fan Mao:** Methodology, Validation, Investigation, Data curation, Writing - original draft, Writing - review & editing, Visualization, Funding acquisition. **Shu Xiao:** Methodology, Validation, Resources, Funding acquisition. **Haiyan Yu:** Methodology, Formal analysis, Investigation, Data curation. **Zhiming Xiang:** Methodology, Validation, Data curation, Funding acquisition. **Fei Xu:** Formal analysis, Validation, Data curation. **Jun Li:** Validation, Resources. **Lili Wang:** Formal analysis. **Yuanyan Xiong:** Formal analysis. **Mengqiu Chen:** Formal analysis. **Yongbo Bao:** Formal analysis. **Yuewen Deng:** Validation. **Quan Huo:** Validation. **Lvping Zhang:** Validation. **Wenguang Liu:** Validation. **Xuming Li:** Formal analysis. **Haitao Ma:** Formal analysis. **Yuehuan Zhang:** Resources. **Xiyu Mu:** Formal analysis. **Min Liu:** Formal analysis. **Hongkun Zheng:** Conceptualization, Formal analysis, Data curation, Project administration. **Nai-Kei Wong:** Writing - review & editing, Visualization. **Ziniu Yu:** Conceptualization, Writing - review & editing, Visualization, Supervision, Project administration, Funding acquisition. All authors have read and approved the final manuscript.

## Competing interests

Haiyan Yu, Lili Wang, Xuming Li, Xiyu Mu, Min Liu, and Hongkun Zheng are current employees of Biomarker Technologies Co., Ltd. All the other authors have declared no competing interests.
